# Effect of acupuncture pretreatment on clinical pregnancy rate in women with diminished ovarian reserve undergoing IVF-ET: study protocol for a multicenter randomized controlled trial

**DOI:** 10.3389/fendo.2026.1723278

**Published:** 2026-02-05

**Authors:** Xin Liu, Chenchen Su, Junmin Li, Li Chen, Qinhua Zhang, Yan Sun, Jinbang Xu, Cuilian Zhang, Xinjie Cheng, Xuemei Li, Huisheng Yang, Yicong Xie, Jie Yang, Na Zhu, Weiying Lu, Tongsheng Su, Wei Shang, Qun Lu, Li Yang, Jiashan Li, Tian Hang, Lingyu Qi, Qiwei Xiao, Weixin Li, Feng Gao, Xiaoyan Wang, Pengfei Du, Dongxue An, Huanfang Xu, Yigong Fang

**Affiliations:** 1 The Institute of Acupuncture and Moxibustion, China Academy of Chinese Medical Sciences, Beijing, China; 2 Reproductive Medicine Center, The Third Affiliated Hospital of Zhengzhou University, Zhengzhou, China; 3 Reproductive Medicine Center, General Hospital of Eastern Theater Command, Nanjing, China; 4 Reproductive Medicine Center, Shanghai University of Traditional Chinese Medicine Affiliated Shuguang Hospital, Shanghai, China; 5 Reproductive Medicine Center, Fujian Maternal and Child Health Hospital, Fuzhou, China; 6 Department of Traditional Chinese Medicine, Fujian Maternal and Child Health Hospital, Fuzhou, China; 7 Reproductive Medicine Center, Henan Provincial People's Hospital, Zhengzhou, China; 8 Department of Rehabilitation Medicine, Henan Provincial People's Hospital, Zhengzhou, China; 9 Reproductive Medicine Center, Shenzhen Maternal and Child Health Hospital, Shenzhen, China; 10 Department of Acupuncture and moxibustion Massage, Shenzhen Maternal and Child Health Hospital, Shenzhen, China; 11 Department of Traditional Chinese Medicine, Tianjin Medical University General Hospital, Tianjin, China; 12 Acupuncture and Tuina School, Chengdu University of Traditional Chinese Medicine, Chengdu, China; 13 Clinical Research Center for Acupuncture and Moxibustion in Sichuan Province, Sichuan Jinxin Xinan Women and Children Hospital, Chengdu, China; 14 Reproductive Medicine Center, Reproductive and Genetic Hospital of China International Trust and Investment Corporation (CITIC)-Xiangya, Changsha, China; 15 Reproductive Medicine Center, Hainan Women and Children's Medical Center, Haikou, China; 16 Department of Acupuncture, Shaanxi Provincial Hospital of Chinese Medicine, Xian, China; 17 Reproductive Medicine Center, The Seventh Medical Center of the Chinese PLA General Hospital, Beijing, China; 18 Reproductive Medicine Center, Beijing Chaoyang Hospital of Capital Medical University, Beijing, China

**Keywords:** acupuncture, clinical pregnancy rate, diminished ovarian reserve, *in vitro*fertilization-embryo transfer, multi-center, randomized controlled trial, study protocol

## Abstract

**Introduction:**

Diminished ovarian reserve (DOR) is known to reduce the likelihood of achieving pregnancy or live births in women undergoing *in vitro* fertilization and embryo transfer (IVF-ET). Acupuncture maybe effective for DOR, but current evidence remains limited and inconclusive.

**Methods and analysis:**

This study is a multicenter, randomized, placebo-controlled trial conducted on a large scale. A total of 300 women with DOR preparing for IVF-ET will be randomized 1:1 to acupuncture or placebo acupuncture. Interventions will be administered from the second menstrual cycle preceding the IVF cycle until the day of oocyte retrieval. The primary outcome is the clinical pregnancy rate (CPR) following the first embryo transfer. Secondary outcomes include various IVF-ET indicators, such as the number of follicles ≥ 14 mm, estradiol (E2) levels, and endometrial thickness on the day of human chorionic gonadotropin (hCG) administration; the number of retrieved oocytes, metaphase II (MII) oocytes, and two pronuclei (2PN) fertilizations; as well as the rates of 2PN fertilization, available embryos, high-quality embryos, implantation, cycle cancellation, biochemical pregnancy, pregnancy loss, sustained pregnancy, and live birth. Additionally, ovarian reserve indicators—including antral follicle count (AFC), basal serum levels of follicle-stimulating hormone (FSH), luteinizing hormone (LH), and E2, and anti-Müllerian hormone (AMH)—along with scores from the Self-Rating Anxiety Scale (SAS) will also be evaluated.

**Ethics and dissemination:**

The trial has been approved by the ethics committees of all participating centers. The results will be disseminated in academic journals.

**Clinical Trial Registration:**

http://itmctr.ccebtcm.org.cn/, identifier ID: ITMCTR2024000021.

## Introduction

Diminished ovarian reserve (DOR) is a momentous contributing factor of female infertility, defined by a reduction in oocyte quantity and/or quality, its diagnostic biochemical profile typically includes elevated serum follicle-stimulating hormone (FSH), alongside decreased levels of anti-Müllerian hormone (AMH) and a less antral follicle count (AFC) (1). The pathogenesis of DOR is associated with multiple factors, including age, genetic, iatrogenic, autoimmune, infectious agents, environmental, and social-psychological (2). Epidemiological data from 2004–2011 showed DOR prevalence rising from 19% to 26% (3). DOR usually leads to unsatisfactory outcomes in *in vitro* fertilization-embryo transfer (IVF-ET). DOR patients achieve lower clinical pregnancy rates (CPR) and live birth rates (LBR) during IVF-ET than those without DOR (4, 5).

The subpar response of DOR patients to exogenous gonadotropins poses a major challenge in the field of reproductive endocrinology (6). Elderly individuals and those with elevated basal FSH levels often display poor responsiveness to ovulation induction due to their diminished ovarian reserve (6). Although various interventions, including growth hormone (GH) and dehydroepiandrosterone (DHEA), have shown potential to optimize ovarian performance and reproductive outcomes in assisted reproduction (6–8). Presently, the effectiveness of these treatment modalities has not been definitively confirmed due to insufficient robust clinical trials.

Acupuncture is regarded as a beneficial alternative therapy for DOR (2). Although further high-quality studies are warranted, accumulating evidence demonstrates that acupuncture effectively modulates reproductive hormone profiles (9), enhances ovarian reserve function (10, 11), increases CPR (10, 11), and reduces anxiety (12) in women undergoing IVF-ET. Given these beneficial effects on ovarian reserve parameters and IVF-ET outcomes, acupuncture represents a promising therapy for DOR.

Accordingly, we initiated a prospective, multicenter, randomized controlled trial to rigorously assess both the efficacy and safety of acupuncture for DOR patients undergoing IVF-ET. The primary outcome is the CPR following the first embryo transfer.

## Methods and design

### Study design

This multicenter, randomized, sham-controlled trial aims to compare the effects of verum acupuncture and non-penetrating placebo acupuncture in women with DOR. A target sample of 300 participants will be recruited from 13 Grade A tertiary hospitals strategically distributed across China: three in both East and North China, three in Central China, two in South China, and one each in the Southwest and Northwest. Participating centres are Sichuan Jinxin Xinan Women and Children Hospital, Shaanxi Provincial Hospital of Chinese Medicine, Hainan Women and Children’s Medical Center, Fujian Maternal and Child Health Hospital, General Hospital of Eastern Theater Command, Tianjin Medical University General Hospital, Reproductive and Genetic Hospital of CITIC-Xiangya, Henan Provincial People’s Hospital, The Third Affiliated Hospital of Zhengzhou University, Shanghai University of Traditional Chinese Medicine Affiliated Shuguang Hospital, Shenzhen Maternal and Child Health Hospital, The Seventh Medical Center of the Chinese PLA General Hospital, Beijing Chaoyang Hospital of Capital Medical University.

**Figure 1:**
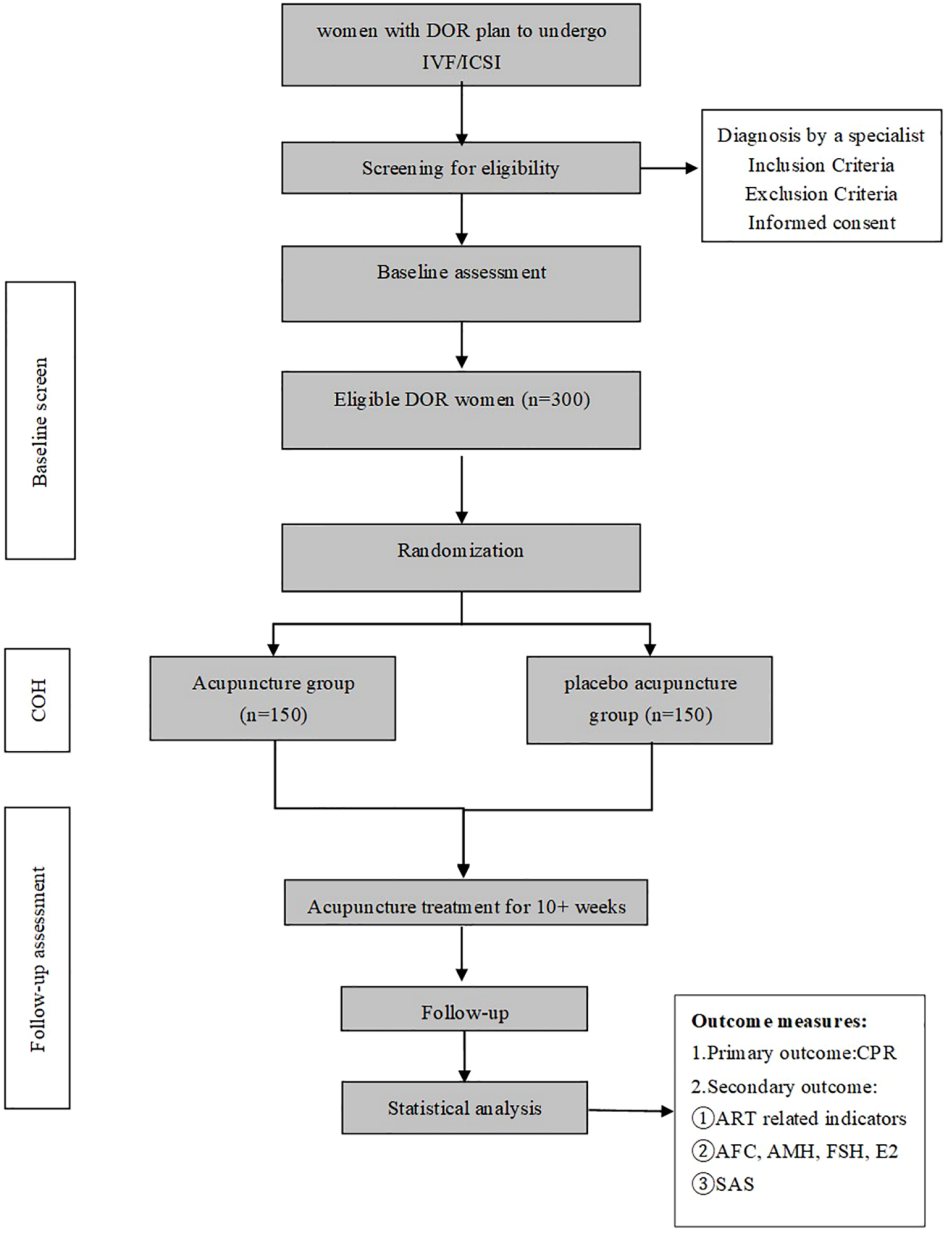
Flow diagram of the trial. DOR, diminished ovarian reserve; IVF, *in vitro* fertilization; ICSI, intracytoplasmic sperm injection; COH, controlled ovarian hyperstimulation; ART, assisted reproductive technology; CPR, clinical pregnancy rate; AFC, antral follicle count; AMH, anti-Müllerian hormone; FSH, follicle-stimulating hormone; E2, estradiol; SAS, Self-Rating Anxiety Scale.

The study received ethical approval from all participating institutions and was prospectively registered at the International Traditional Medicine Clinical Trial Registry (registration number: ITMCTR2024000021) on 21 February 2024. Participant progression throughout the trial is depicted in the CONSORT-compliant flowchart (**Figure 1**), and key procedural timelines aligned with the SPIRIT checklist are summarized in **Table 1**.

**Table 1:** Schedule of recruitment, intervention, and measures.

Time Items	Visit 1	Visit 2	Visit 3	Visit 4	Visit 5
	Screening period (-8 to 0 weeks)	Intervention period (+4 weeks)	Intervention period (+8 weeks)	Intervention period/COH cycle (+10 weeks)	Follow-up (+24 weeks)
Informed consent	**×**				
Inclusion/Exclusion Criteria	**×**				
Demographic data	**×**				
Medical history	**×**				
Treatment history	**×**				
Comorbidities	**×**				
Acupuncture/Placebo acupuncture		**×**	**×**	**×**	
AFC	**×**			**×**	
FSH, LH, E2	**×**			**×**	
AMH	**×**			**×**	
SAS	**×**			**×**	
IVF/ICSI				**×**	
Pregnancy outcomes					**×**
Concomitant medication		**×**			
Adverse event		**×**			

COH, controlled ovarian hyperstimulation; AFC, antral follicle count; FSH, follicle-stimulating hormone; LH, luteinizing hormone; E2, estradiol; AMH, Anti-Mullerian hormone; SAS, Self Rating Anxiety Scale; IVF, *in vitro* fertilization; ICSI, intracytoplasmic sperm injection.

### Participants

Potential participants will be identified by dedicated clinicians through hospital-based advertisements (printed posters, WeChat official accounts, and approved online platforms). Before enrollment, a qualified clinician will administer a detailed briefing to each eligible participants, covering the trial’s objectives, methodology, procedures, anticipated benefits, and potential risks. Written informed consent will be formally documented from all participants prior to the initiation of any trial-related activities.

#### Inclusion criteria

Women meeting the diagnostic criteria for DOR (13) and scheduled for IVF-ET will be enrolled provided they fulfill all the following conditions:

1. Age 20–39 years;
2. AFC<5, AMH<1.2 ng/mL (13);
3. With regular menstrual cycles (21–35 days);
4. With GnRH antagonist or microstimulation protocol for controlled ovarian hyperstimulation (COH);
5. Plan to transfer 1–2 high-quality cleavage embryos or one blastocyst;
6. Able and willing to give written informed consent.

#### Exclusion criteria

We will exclude individuals meeting any of the following criteria:

1. Basal FSH ≥ 25 IU/L;
2. Congenital malformations of the reproductive tract; untreated endometrial polyps, intrauterine adhesions, active uterine infection, or hydrosalpinx; endometrial thickness < 7 mm on the HCG trigger day; submucosal fibroids > 5 cm in diameter; or any stage of endometriosis;
3. Repeated implantation failure, defined as failure to achieve clinical pregnancy after ≥2 fresh or frozen ET cycles with the transfer of ≥4 top-grade cleavage embryos or ≥2 high-quality blastocysts;
4. Hyperprolactinaemia, hyperandrogenism, thyroid dysfunction, chronic adrenal insufficiency, or any other endocrine or metabolic disorder that has not been normalized with standard therapy;
5. Male partner with azoospermia;
6. Severe cardiovascular, cerebrovascular, hepatic, renal, malignant, haematological, or psychiatric disease;
7. Acupuncture for infertility within the preceding 3 months;
8. Recurrent pregnancy loss, defined as ≥ 3 consecutive spontaneous miscarriages < 28 weeks’ gestation with the same partner;
9. Clinically significant anxiety, indicated by a Self-Rating Anxiety Scale (SAS) score > 70;
10. Chromosomal abnormalities in either partner.

#### Randomization method

Randomization will be performed after obtaining informed consents. A secure online computerized randomization system will be utilized for randomization to ensure allocation concealment. This centralized web-based platform incorporates multiple functional modules for randomization, data entry, and management, and will be overseen by an independent statistical team. Qualified participants will be assigned to the acupuncyure or placebo acupuncyure groups in a 1:1 allocation ratio, employing stratified block randomization by age (< 35 years vs. ≥ 35 years) through a computer-generated system. Upon enrollment of eligible subjects, personnel responsible for randomization will log into the central randomization system via the internet to obtain a randomization number.

#### Blinding

Blinding procedures will be implemented for study participants, efficacy evaluators, and statistical analysts. Given the inherent characteristics of acupuncture, there is no blindness for acupuncturists. The acupuncturists will not participate in any baseline assessment, randomization procedures, or outcome evaluation of the participants.

### Intervention

All acupuncture procedures will be performed by licensed acupuncturists possessing at least three years of clinical experience. Prior to the initiation of the trial, all participating acupuncturists will complete a comprehensive training program, which will focus on standard operating procedures for acupuncture, placebo acupuncture protocols. Detailed and quantifiable standardized operational procedures (SOPs) will be established for both the acupuncture and placebo acupuncture groups. These SOPs specified the acupoint localization methods, needling angle and depth, needle manipulation techniques, and needle retention time. Independent quality controllers will be designated to regularly assess acupuncturists’ adherence to the SOPs through on-site observation or video recording.

The acupuncture group will utilize sterile disposable needles (Hwato, Suzhou, China) of the following dimensions: 0.25×25 mm, 0.25×40 mm, and 0.30×75 mm. The placebo acupuncture group will receive non-penetrative placebo acupuncture using blunt-tipped needles (0.25×25 mm; Hwato, Suzhou, China), which are specifically engineered to mimic the tactile sensation of real needle insertion while preventing actual skin penetration. Both groups will utilize an adhesive circular pad with a diameter of 1 cm. This pad serves dual purposes: securing the placebo needle to the skin surface and facilitating the blinding process. Following standard skin disinfection, each acupoint will be covered with a sterile pad that directs the placebo needle to the skin surface. Depending on the group assignment as per the study protocol, the needle will either be inserted to a predetermined depth (acupuncture group) or remain on the skin surface without penetration (placebo acupuncture group).

#### Acupuncture group

For the acupuncture group, two sets of acupoints (Set 1 and Set 2) will be used: Set 1 and Set 2. Set 1 (administered in the supine position) includes the following points: CV12 (Zhongwan), KI16 (Huangshu), CV4 (Guanyuan), KI12 (Dahe), SP6 (Sanyinjiao), and LR3 (Taichong). Set 2 (administered in the prone position) consists of BL23 (Shenshu), BL33 (Zhongliao), and KI3 (Taixi). The two sets will be alternated, with Set 1 used for the initial treatment. Details regarding the anatomical locations of these acupoints and the specific manipulation techniques employed in the acupuncture group are presented in **Table 2**. All acupoint locations will be determined in compliance with the World Health Organization Standardized Acupuncture Points guidelines (14).

**Table 2:** Location of acupoints and manipulation techniques used for Acupuncture group.

Acupoints	Location	Manipulation
Set 1 acupoints		
CV12 (Zhongwan)	On the anterior midline, 4 cun above the umbilicus	Inserted vertically to a depth of 1.0 cun–1.5 cun
KI16 (Huangshu)	Bilateral,0.5 cun lateral to the umbilicus	Inserted vertically to a depth of 1.0 cun–1.5 cun
CV4 (Guanyuan)	On the anterior midline, 3 cun below the umbilicus	Inserted vertically to a depth of 1.0 cun–1.5 cun
KI12 (Dahe)	Bilateral, 4 cun below the umbilicus, and 0.5 cun lateral to the lower anterior midline	Inserted vertically to a depth of 1.0 cun–1.5 cun
SP6 (Sanyinjiao)	Bilateral, posterior to the medial border of the tibia, and 3 cun above to the tip of the medial malleolus	Inserted vertically to a depth of 1.0 cun–1.5 cun
LR3 (Taichong)	Bilateral, in the depression anterior to the junction of the first and second metatarsal bones	Inserted vertically to a depth of 0.5 cun– 0.8 cun
Set 2 acupoints		
BL23 (Shenshu)	Bilateral, 1.5 cun lateral to the depression below the spinous of the second lumbar vertebra	Inserted vertically to a depth of 1.0 cun–1.5 cun
BL33 (Zhongliao)	Bilateral, in the sacral region, and precisely at the site of the third sacral posterior foramen	inserted bilaterally into the third posterior sacral foramina (at BL33) and angled inwards and downwards at 50–60° to a depth of 60–70 mm
KI3 (Taixi)	Bilateral, in the depression between the tip of the midial malleolus and the Achilles tendon	Inserted vertically to a depth of 0.5 cun–1.0 cun

1 cun (≈ 20 mm) is defned as the width of the interphalangeal joint of the participant’s thumb.

Participants will assume the appropriate body position corresponding to the designated set of acupoints. After disinfecting the skin, sterile adhesive pads will be positioned at each acupoint, through which acupuncture needles will be advanced into the skin. Following needle insertion, standardized manual techniques (lifting, thrusting, and twirling) will be applied to elicit deqi, a characteristic complex of needling sensations including soreness, numbness, distension, or heaviness considered essential for therapeutic efficacy (15). Each acupuncture session will last 20 minutes.

**Figure 2:**
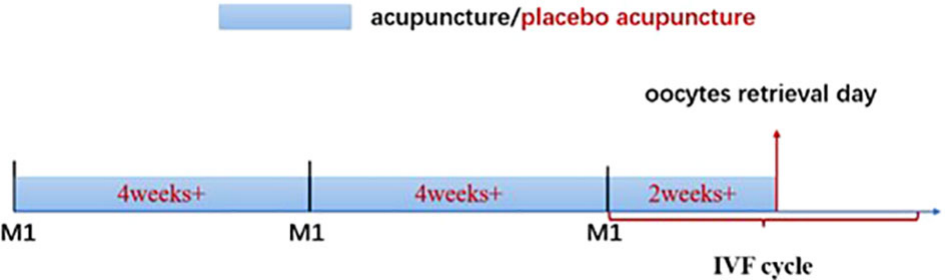
Diagram of acupuncture treatment course. M1, on the first day of the menstrual cycle; IVF, *in vitro* fertilization.

Acupuncture interventions will commence during the first two menstrual cycles preceding the IVF cycle and continue until the day of oocyte retrieval (**Figure 2**). Each participant will complete three sessions weekly (ideally spaced every other day) over roughly ten consecutive weeks, about 30 sessions. If menstruation occurs during the acupuncture treatment period, the acupuncture sessions will proceed as scheduled without interruption.

#### Placebo acupuncture group

For the placebo acupuncture group, two sets of acupoints (Set 1 and Set 2) will also be used. To reduce specific therapeutic responses, non-acupoint sham sites distant from established acupoints will be utilized, with detailed locations and stimulation parameters documented in **Table 3**. The selection of sham acupoints for the control group is based on the “non-meridian, non-acupoint” principle. Points are located on the body surface at least 1 cun (a traditional unit of measurement, about 20 mm) away from any true acupoint and not situated on a recognized meridian pathway. Moreover, a non-penetrating sham needle is used at these locations to further minimize physiological stimulation. This combined approach (employing both non-meridian, non-acupoint sites and a non-penetrating needle technique) has been reported previously (16) and is considered an effective method for blinding participants and reducing nonspecific therapeutic effects.

**Table 3:** Location of points and manipulation techniques used for placebo acupuncture group.

Acupoints	Location	Manipulation
Set 1 acupoints		
Non-acupoint 1	On the anterior midline, 4 cun above the umbilicus, 1 cun to the left from CV12 (Zhongwan)	No skin penetration
Non-acupoint 2	Bilateral, 1.5 cun lateral to the umbilicus, 1 cun lateral to KI16(Huangshu)	No skin penetration
Non-acupoint 3	On the anterior midline, 3 cun below the umbilicus, 1 cun to the right from CV4 (Guanyuan)	No skin penetration
Non-acupoint 4	Bilateral, 4 cun below the umbilicus, and 1.5 cun lateral to the lower anterior midline, 1 cun lateral to KI12(Dahe)	No skin penetration
Non-acupoint 5	Bilateral, 3 cun above the tip of the medial malleolus between SP6 (Sanyinjiao) and Achilles tendon	No skin penetration
Non-acupoint 6	Bilateral, in the depression anterior to the junction of the second and third metatarsal bones	No skin penetration
Set 2 acupoints		
Non-acupoint 7	Bilateral, 0.75 cun lateral to BL23(Shenshu)	No skin penetration
Non-acupoint 8	Bilateral, 1 cun lateral to BL33(Zhongliao)	No skin penetration
Non-acupoint 9	Bilateral, on the inner side of the foot, behind the inner ankle, at the same level as KI3 (Taixi), at the anterior edge of the Achilles tendon	No skin penetration

1 cun (≈ 20 mm) is defned as the width of the interphalangeal joint of the participant’s thumb.

The two sets will be alternated, with Set 1 used as the initial treatment. Placebo acupuncture will be administered using blunt-tip placebo needles (Hwato, 0.25×25 mm) that prevent cutaneous penetration. Consistent with the acupuncture group, adhesive pads will initially be applied to the surface of placebo acupoints, and blunt-tipped pragmatic placebo needles will be placed on these pads. To minimize physiological effects, acupuncturists will follow standardized procedures for positioning blunt-tipped placebo needles on adhesive pads, maintaining light contact while avoiding any manipulative techniques. Placebo needles will be positioned superficially at control points using a non-penetrative technique that prevents actual skin puncture. The needle retention time and treatment course will be identical to the acupuncture group.

Throughout the trial, participants will receive isolated treatments to limit cross-communication and will be advised to refrain from concurrent relevant therapies. Any such additional treatments received will require documentation.

#### COH regimen

COH will be administered starting on menstrual cycle days 2–3 after pretreatment completion, utilizing either a GnRH antagonist protocol or microstimulation protocol in all enrolled participants. The administration of both GnRH antagonist and microstimulation protocols will be managed by certified reproductive endocrinology specialists across all trial sites. As some participants may meet the criteria for IVF initiation before concluding the full acupuncture pretreatment, this study will permit the initiation of COH cycles provided that patients have completed a minimum of 8 weeks of acupuncture pretreatment.

#### Permitted and prohibited concomitant treatments

To ensure unbiased evaluation of acupuncture outcomes, participants will be advised to avoid concomitant interventions such as traditional Chinese herbs, Chinese patent medicines, growth hormone preparations. Any form of acupuncture outside the experimental protocol will be prohibited.

### Outcome measurement

#### Primary outcome

The primary outcome is the CPR following the first fresh or frozen embryo transfer. Ultrasonographic identification of a gestational sac and fetal heartbeat at 28 days post-embryo transfer confirmed a clinical pregnancy. The CPR is defined as the proportion of clinical pregnancies to the total number of embryo transfer cycles.

#### Secondary outcomes

The secondary outcomes include the number of follicles ≥ 14 mm, E2 level, and endometrial thickness on the hCG trigger day; the number of retrieved oocytes, metaphase II (MII) oocytes, and two pronuclei (2PN) fertilizations; as well as the rates of 2PN fertilization, available embryos, high-quality embryos, implantation, cycle cancellation, biochemical pregnancy, pregnancy loss, sustained pregnancy, and live birth. The outcomes of IVF-ET and their definitions are summarized in **Table 4**. While the post-treatment observation will be terminated at about 24 weeks, data on live birth will be obtained through the participating centers’ mandatory, complete follow-up system extending to the end of pregnancy.

**Table 4:** Diagnosis definitions of IVF-ET outcomes.

Diagnosis	Definition
Primary outcomes	
clinical pregnancy rate (CPR)	the number of clinical pregnancies divided by the total number of embryo transfer cycles.
Secondary outcomes	
Outcomes of IVF-ET	
2PN fertilization rate	the number of 2PN embryos divided by the number of MII oocytes
available embryo rate	the number of transferable embryos divided by the number of 2PN fertilization
high-quality embryo rate	the number of high-quality embryos divided by the number of normally fertilized cleavage embryos. High-quality embryos were defined based on the day of embryo transfer, according to the Istanbul consensus and Gardner criteria, as follows: Day 2: embryos with 4 cells, <10% fragmentation, and absence of multi-nucleation; Day 3: embryos with 8 cells, <10% fragmentation, and no multi-nucleation; Day 5: blastocysts at stage 4, with inner cell mass and trophectoderm both graded as A (17, 18).
Implantation rate	the number of gestational sacs divided by the number of embryos transferred.
cycle cancellation rate	the number of cancelled embryo transfer cycles divided by the number of embryo transfer cycles
biochemical pregnancy rate	the number of patients with positive serum b-hCG pregnancy test results divided by total embryo transfer cycles
pregnancy loss rate	the number of pregnancy termination cycles within 12 weeks divided by the number of embryo transfer cycles
sustained pregnancy rate	the number of cycles of pregnancy greater than 12 weeks divided by the number of embryo transfer cycles
live birth rate	the number of live births divided by total embryo transfer cycles.
Scale measurement	
self-rating anxiety scale (SAS)	SAS consists of 20 items, and its measurement is determined by calculating the sum of the scores of each item

Additionally, ovarian reserve will be assessed using AFC and serum levels of AMH, FSH, LH, and E2. AFC and serum levels of FSH, LH, and E2 will be measured on days 2 to 5 of the menstrual cycle, while AMH can be measured on any day of the menstrual cycle. These assessments will be conducted both before and after the acupuncture intervention, with the post-intervention assessment scheduled during the menstrual period of the IVF cycle.

Anxiety levels will be evaluated using the Self-Rating Anxiety Scale (SAS). The total SAS score reflects the severity of anxiety symptoms, with a standard cutoff score of 50 used to classify participants into four categories: no anxiety (<50), mild anxiety (50–59), moderate anxiety (60–69), and severe anxiety (≥70) (19). The SAS will be performed before the first acupuncture session and within 24 hours after the final session.

### Blinding assessment

Upon completion of the full acupuncture treatment, participants in both the two groups will be surveyed on the oocyte retrieval day to guess their group assignment, thereby testing the blinding integrity.

### Assessment of safety

Adverse events (AEs) will be systematically monitored and recorded throughout the trial. AEs will be classified by severity (mild, moderate, severe) and causality (divided into 5 levels: definitely unrelated, probably unrelated, possibly related, probably related, definitely related) (20). All events will be documented in the case report form (CRF) with details on the exact time of onset, interval from needling, manifestations, management, and outcome, with causality judged by the acupuncturist.

Acupuncture-related AEs, potentially including hematoma, bleeding, subcutaneous hemorrhage, severe pain, local infection, and fatigue, will be collected at each treatment session. AEs associated with IVF-ET encompass pain, organ injury, colporrhagia, infections secondary to oocyte retrieval procedures, ovarian hyperstimulation syndrome (OHSS), thrombosis, allergic reactions, post-transplant abdominal pain, breast swelling, frequent urination, and nausea. All AEs will be characterized by the principal investigator and an independent data-safety monitor in terms of severity, causality, seriousness, and outcome, with serious ones requiring immediate reporting to the institutional review board within 24 hours.

### Data management

This project employs a web-based data management system for data entry. Prior to data collection, data entry personnel will receive specialized training based on a predefined Data Entry Guideline. All entered data will undergo rigorous verification according to the Data Validation Plan, utilizing both automated system checks and manual medical reviews. Any database modifications are fully documented with timestamps, personnel identification, and rationale. Before database lock, the data management unit prepares a comprehensive Data Management Report for sponsor review and approval. During the Data Review Meeting, stakeholders (data managers, statisticians, investigators) discuss critical elements—including subject disposition, protocol deviations, potential outliers, baseline characteristics, efficacy endpoints, and finalization of the Statistical Analysis Plan (SAP)-based on the Data Management Report and data listings, culminating in data review resolutions. The locked database is delivered to statisticians in SAS transport files (.xpt format) accompanied by dataset specifications and codebooks for statistical analysis.

#### Quality control

The development of the protocol has been informed by iterative refinement and critical evaluation from a multidisciplinary team of acupuncture, methodological, and statistical experts. We developed comprehensive Standard Operating Procedures (SOPs) covering intervention delivery, Case Report Form (CRF) completion, outcome assessment, and data management. All personnel received standardized training to ensure protocol compliance. A comprehensive quality control system will be implemented, including random patient verification via telephone audits and spot checks of researchers’ informed consent documentation. Acupuncture protocol fidelity will be prioritized, with monitoring of: Practitioner certification for this trial, Acupoint localization accuracy, Sterilization compliance, Needling depth/technique standardization, Manipulation consistency.Verification methods will include direct observation, simulated procedure assessments, and practitioner interviews.

#### Sample size

Based on previous research (21, 22), we estimated that the CPR of IVF-ET in DOR patients after acupuncture pretreatment would be 38%, compared to 22% following placebo acupuncture pretreatment. A sample size of 120 participants per group has been determined to ensure 80% statistical power, with a two-sided alpha of 0.05. An initial total of 300 participants (150 per group) will be enrolled to allow for an anticipated 20% dropout rate.

#### Statistical analysis

Analyses will be performed using SAS version 9.3 (SAS Institute Inc., Cary, NC, USA). The intention-to-treat (ITT) principle will be applied to all data analyses in this study. Multiple imputation will be employed to manage missing data. Continuous variables will be reported as mean ± standard deviation or median (interquartile range), and categorical variables as frequencies (percentages).

The primary outcome, CPR, was analyzed using multivariate logistic regression to calculate adjusted odds ratios (aORs) with 95% confidence intervals (CIs). The dependent variable in the model was clinical pregnancy (yes/no), and the independent variable was group allocation. The selection of covariates for adjustment was based on their established clinical relevance to IVF outcomes and any observed baseline imbalances between groups (p < 0.10). These covariates may include centers, age, baseline FSH level, AFC, COH protocol, number of retrieved oocytes, type of ET, number of embryos transferred, etc. Model fit was assessed using the Hosmer-Lemeshow test, and multicollinearity was evaluated via variance inflation factors. Additionally, a Per-Protocol (PP) analysis for priamry outcome will be performed as a sensitivity analysis. Other between-group comparisons will be performed using independent t-tests/Wilcoxon rank-sum tests for continuous data and chi-square/Fisher’s exact tests for categorical data, as appropriate. A two-sided *p* < 0.05 will define statistical significance.

## Discussion

This trial is designed to evaluate the efficacy and safety of acupuncture in improving the CPR and ovarian function in women with DOR undergoing IVF-ET.

DOR manifests as a reduction in the number of oocytes and compromised oocyte quality, a reduction in CPR and LBR, and an increase in miscarriage rates (23). In individuals with DOR, the CPR and LBR persist at a low level, regardless of age (4, 23, 24). In the context of ovarian reserve assessment, the primary concern for patients is their ability to achieve pregnancy and ultimately deliver a live birth. Given the extended observation period necessary to evaluate the LBR, we selected the CPR as the primary outcome measure.

Additionally, ovarian reserve testing-related indicators, including AFC and serum levels of AMH, FSH, LH, and E2, will be selected as secondary outcome measures. Ovarian reserve testing provides valuable information for assessing ovarian reserve and reproductive potential, as these factors serve as key determinants of pregnancy outcomes (4). Ovarian reserve is typically evaluated using several indicators, including age, AFC, and levels of FSH and AMH (1). Since pituitary FSH secretion is inhibited by feedback from ovarian factors, the resultant basal FSH level can serve as an indirect gauge of ovarian reserve (25). A rise in FSH levels during the early follicular phase is interpreted clinically as evidence of compromised ovarian hormone production, which is associated with a reduced ovarian follicular pool consistent with DOR (25). However, this test exhibits significant intercycle and intracycle variability, thereby limiting its reliability (26). AMH serves as a direct biomarker for the primordial follicle pool, as it is secreted from the granulosa cells of small, developing follicles. Therefore, serum AMH is considered the preferred biomarker for assessing ovarian reserve,although the absence of an international standard hinders direct comparisons across different assays (27). Serum AMH levels have demonstrated predictive value for clinical pregnancy in IVF-ET cycles among women with a diminished ovarian response (28). The AFC is defined as the total number of antral follicles with a diameter ranging from 2 to 10 mm, observed on ovarian ultrasound during the early follicular phase (1, 29). AFC measurements exhibit good inter-cycle and inter-observer reliability in centers with experienced sonographers (1, 29).

Furthermore, the predominant number of individuals diagnosed with DOR manifest symptoms of anxiety to varying extents. The utilization of the SAS, a tool specifically developed to gauge anxiety and psychological stress, will serve to evaluate the levels of anxiety present in DOR women (30).

Acupuncture has been shown to modulate gonadotropin levels, lowering FSH, LH, and the FSH/LH ratio, while elevating E2 and ameliorating emotional symptoms in DOR patients, with a favorable safety profile (9–11). Research indicated that acupuncture contributes to higher CPR in IVF-ET for DOR women by effectively improving their underlying ovarian reserve function (31). Another investigation demonstrated that acupuncture positively influences hCG-day estrogen levels, endometrial conditions, thereby improved endometrial receptivity during IVF-ET (32). However, existing trials are limited by small sample sizes. This large-sample, multicenter RCT is designed to definitively assess the efficacy and safety of the acupuncture intervention, thereby addressing this critical evidence gap.

According to traditional Chinese medicine (TCM) theory, an imbalance of the Ren Meridian (Conception Vessel, CV) and Chong Meridian (Penetrating Vessel, PC) constitutes a fundamental pathogenesis of DOR, and is associated with kidney deficiency, liver stagnation, and spleen deficiency. Therefore, the main therapeutic principles for DOR involve regulating the Chong and Ren Meridians, tonifying the kidney, regulating the liver and fortifying the spleen. During this clinical trial, the selected acupuncture points will include CV 12 (Zhongwan) and CV 4 (Guanyuan) from the Ren Meridian; KI 12 (Dahe) and KI 16 (Huangshu), which are intersection points of the Kidney Channel of Foot-Shaoyin and the Chong Meridian; SP 6 (Sanyinjiao), which serves to invigorate the kidney, regulate the liver, and fortify the spleen; BL 23 (Shenshu), BL 33 (Zhongliao), and KI 3 (Taixi), which are utilized to reinforce the kidney and nourish the essence; and LR 3 (Taichong), which alleviates liver tension and harmonizes qi.

The methodological rigor of this trial, particularly regarding randomization, allocation concealment, and blinding across all study stages, will be safeguarded by the use of placebo needles, adhesive pads, and an EDC system.

This study possesses several constraints. Initially, it will encompass solely DOR women aged < 40 who are eligible for either GnRH antagonist protocols or microstimulation protocols, which might diminish the representativeness of the sample population. Secondly, owing to the intrinsic characteristics of acupuncture, operator-blinding is not feasible in this trial comparing acupuncture and placebo acupuncture.

In conclusion, this trial employs a rigorous and well-established acupuncture protocol for the treatment of DOR. The results may support acupuncture as a viable treatment for DOR patients.
