# Supplementary material for: Effect of acupuncture pretreatment on clinical pregnancy rate in women with diminished ovarian reserve undergoing IVF-ET: study protocol for a multicenter randomized controlled trial
Source: Front Endocrinol (Lausanne). 2026 Feb 5;17:1723278. doi: 10.3389/fendo.2026.1723278 (PMC12917761; doi:10.3389/fendo.2026.1723278)
Supplement: Supplementary file 1 [file DataSheet1.docx]

**Supplementary Material – Appendix 1**

**Appendix 1 The name of the ethics committees and the ethics approval number.**

| **Name of** **Ethics Committees (EC)** | **Ethics approval number** |
| --- | --- |
| **Team leader unit** | |
| Institute of Acupuncture and Moxibustion, China  Academy of Chinese Medical Sciences | 2024-01-26-6 |
| **Research sub centers (13)** | |
| EC of Sichuan Jinxin Xinan Women and Children Hospital | 2024-09 |
| EC of Shaanxi Provincial Hospital of Chinese Medicine | 2024-18 |
| EC of Hainan Women and Children's Medical Center | 2024-164 |
| EC of Fujian Maternal and Child Health Hospital | 2024KY086-02 |
| EC of General Hospital of Eastern Theater Command | DZQH-KYLL-24-19 |
| EC of Tianjin Medical University General Hospital | IRB2024-YX-057-01 |
| EC of Reproductive and Genetic Hospital of CITIC-Xiangya | LL-SC-2024-019 |
| EC of Henan Provincial People's Hospital | 2024-092 |
| EC of the Third Affiliated Hospital of Zhengzhou University | 2024-185-02 |
| EC of Shanghai University of Traditional Chinese Medicine Affiliated Shuguang Hospital | 2024-1554-137-01 |
| EC of Shenzhen Maternal and Child Health Hospital | SFYLS-2024-093 |
| EC of the Seventh Medical Center of the Chinese PLA General Hospital | S2024-057-01 |
| EC of Beijing Chaoyang Hospital of Capital Medical University | 2024-912-1 |
